# Author’s Response to Peer Review of “Predicting Health Disparities in Regions at Risk of Severe Illness to Inform Health Care Resource Allocation During Pandemics: Observational Study”

**DOI:** 10.2196/25573

**Published:** 2020-12-02

**Authors:** Tara Fusillo

**Affiliations:** 1 John F Kennedy High School Bellmore, NY United States

**Keywords:** coronavirus, SARS-CoV-2, pandemic, socioeconomic status, predictive model, health care resource allocation

 The author of the manuscript [1] is grateful to the editor and reviewers for their invaluable input and feedback.

## Response to Round 1 Reviews

### Specific Comments

#### Major Comments

- The author has added a correlation matrix to the supplemental materials.
- The author has added several examples of stepwise regression model use to the Introduction section to help ground readers in the validity of this approach in similar applications.
- The author has made the suggested points in the revised Limitations section of the Discussion.

#### Minor Comments

- All data sets and regression model details have been added for each state to the Multimedia Appendices section.
- The author has changed the conditional formatting to red and blue in Figure 3.
